# Supplementary material for: Development of Immunochromatographic Test Kit for Rapid Detection of Specific IgG4 Antibody in Whole-Blood Samples for Diagnosis of Human Gnathostomiasis
Source: Diagnostics (Basel). 2021 May 11;11(5):862. doi: 10.3390/diagnostics11050862 (PMC8151850; doi:10.3390/diagnostics11050862)
Supplement: Supplementary file 1 [file diagnostics-11-00862-s001.zip › diagnostics-1198345-supplementary.pdf]

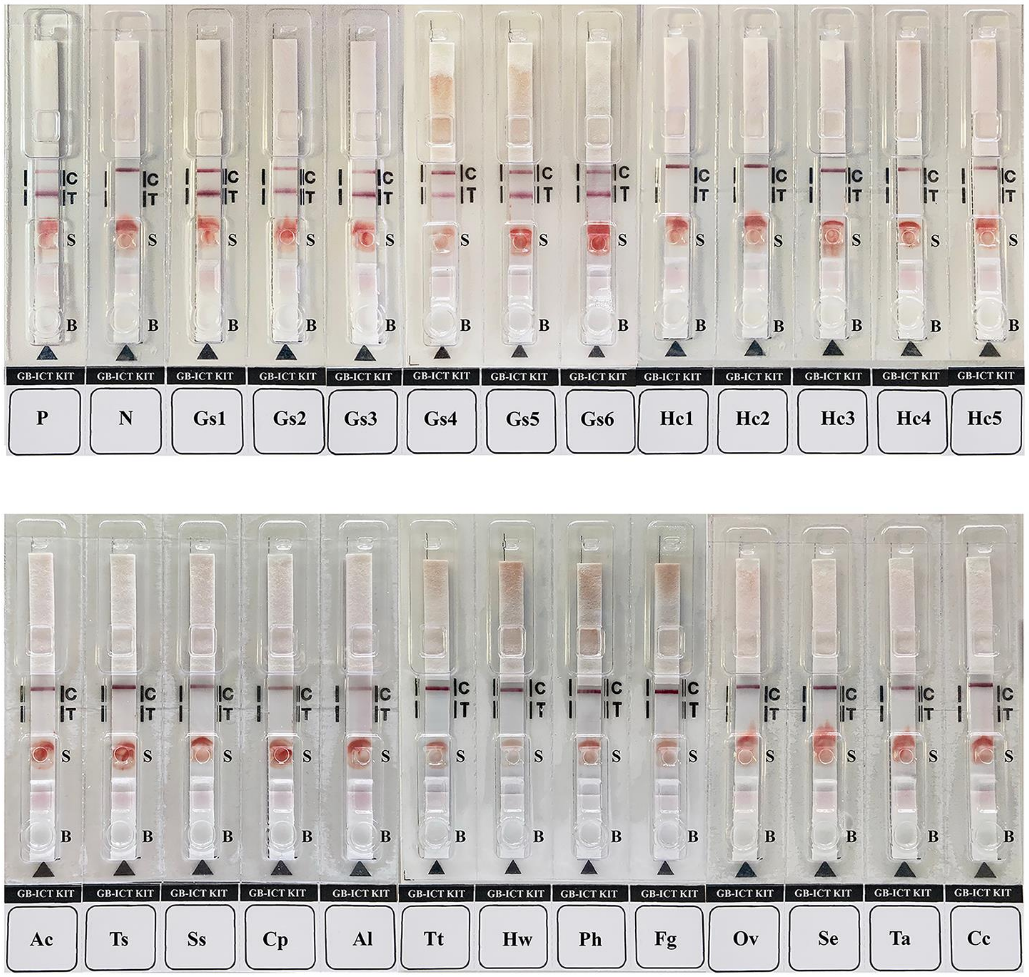

Figure S1. Representative images of anti-*Gnathostoma* IgG4 antibody detected in simulated whole-blood samples (WBSs) by the gnathostomiasis blood immunochromatographic test (GB- ICT) kit. C, control line; T, test line; S, sample well; B, buffer well; P, pooled positive control; N, pooled negative control; Gs1–Gs6, gnathostomiasis; Hc1–Hc5, healthy volunteers; Ac, angiostrongyliasis cantonensis; Ts, trichinellosis spiralis; Ss, strongyloidiasis; Cp, capillariasis philippinensis; Al, ascariasis; Tt, trichuriasis; Hw, hookworm infections; Ph, paragonimiasis heterotremus; Fg, fascioliasis gigantica; Ov, opisthorchiasis viverrini; Se, sparganosis; Ta, taeniasis saginata; Cc, cysticercosis.
